# Editorial: The microbiome-gut-brain axis and posttraumatic stress disorder

**DOI:** 10.3389/fnins.2025.1592604

**Published:** 2025-04-07

**Authors:** Robin M. Voigt, Christopher A. Lowry

**Affiliations:** ^1^Rush Center for Integrated Microbiome and Chronobiology Research, Rush University, Chicago, IL, United States; ^2^Department of Internal Medicine, Rush University Medical Center, Chicago, IL, United States; ^3^Department of Integrative Physiology, University of Colorado Boulder, Boulder, CO, United States

**Keywords:** microbiome-gut-brain axis (MGBA), microbiome, post traumatic stress disorder (PTSD), dysbiosis, short chain fatty acid (SCFA), psychobiotics, neuroinflammation, holistic treatment

The complex interactions between the gut microbiome and the brain, known as the microbiome-gut-brain axis, have emerged as a critical frontier in understanding and potentially treating complex psychiatric disorders, such as posttraumatic stress disorder (PTSD). This special topic consolidates a spectrum of research, revealing a compelling narrative: the gut is a vital communication hub, influencing mental health and offering novel therapeutic avenues for those suffering from the lingering effects of trauma.

Disruption of the gut microbiota, or dysbiosis, emerges as a central theme across these studies. Specifically, research consistently reveals that individuals with PTSD exhibit distinct gut microbial signatures compared to trauma-exposed controls. These signatures are often characterized by reduced microbial diversity and shifts in the abundance of specific bacterial taxa, with some demonstrating a direct correlation to PTSD symptom severity. For instance, Winder et al.'s systematic review, “*Gut microbiome differences in individuals with PTSD compared to trauma-exposed controls*,” provides compelling evidence of this link, underscoring the necessity for further investigation into the underlying causal mechanisms.

One of the most promising areas of investigation into the role of the microbiome-gut-brain axis in responses to, and recovery from, trauma and stressors, is the role of short-chain fatty acids (SCFAs), which are metabolic byproducts of gut microbial fermentation. These compounds, including acetate, propionate, and butyrate, act as crucial signaling molecules, influencing neuroinflammation and brain function. Studies within this Research Topic suggest that imbalances in SCFA production, particularly reduced propionate levels, may contribute to PTSD pathophysiology. This is supported by findings in “*Prebiotics as an adjunct therapy for posttraumatic stress disorder: a pilot randomized controlled trial*” (Voigt et al.) and further explored in “*Molecular mechanisms and therapeutic possibilities of short-chain fatty acids in posttraumatic stress disorder patients: a mini-review*” (Petakh et al.), highlighting the potential of microbiota-modifying interventions, such as prebiotics and probiotics, to augment SCFA production and alleviate PTSD symptoms.

Beyond specific metabolites, the research in this special topic underscores the importance of a holistic approach to PTSD treatment. Traditional therapies, while effective for some, often fail to address the intricate interplay between physiological and psychological factors. This special topic illuminates the need to consider the impact of chronic stress, neuroinflammation, and lifestyle factors on the microbiome-gut-brain axis. “*Using lifestyle interventions and the gut microbiota to improve PTSD symptoms*” (Sugden and Merlo) advocates for lifestyle changes as a vital part of a holistic treatment approach. Lifestyle interventions, including dietary modifications and the use of prebiotics, emerge as potential adjunct therapies for current treatment approaches, offering a more comprehensive strategy for symptom management.

Furthermore, the exploration of novel therapeutic targets extends beyond conventional probiotics and prebiotics. A preclinical study investigates the effects of *Mycobacterium aurum* and demonstrates the potential of mycobacteria to modulate the gut microbiome and mitigate the negative effects of chronic stress. This is demonstrated in “*Effects of repeated intragastric administrations with heat-inactivated Mycobacterium aurum DSM 33539 on the stress-induced aggravation of dextran sulfate sodium (DSS) colitis in C57BL/6N mice*” (Langgartner et al.), opening up exciting possibilities for the development of new psychobiotic interventions, harnessing the immunomodulatory properties of diverse microbial species.

The use of advanced methodologies, such as Mendelian randomization, provides valuable insights into the causal relationships between gut microbiota, psychiatric disorders, and related comorbidities like irritable bowel syndrome (IBS). These studies reveal that genetic predispositions to PTSD and other psychiatric conditions can influence gut microbial composition and increase the risk of IBS, highlighting the shared pathophysiological pathways. This is shown in “*Genetic associations and potential mediators between psychiatric disorders and irritable bowel syndrome: a Mendelian randomization study with mediation analysis*” (Zhang et al.).

Finally, “*The importance of the gut microbiome and its signals for a healthy nervous system and the multifaceted mechanisms of neuropsychiatric disorders*” (Riehl et al.) provides a comprehensive overview of the communication between the gut microbiota and the brain, emphasizing the need for a multidisciplinary approach to understand and treat PTSD. By integrating research from microbiology, neuroscience, psychiatry, and genetics, we can unravel the complex interplay between the gut microbiome and the brain.

In essence, this special topic illuminates the pivotal role of the microbiome-gut-brain axis in the pathophysiology and potential treatment of PTSD. From identifying distinct microbial signatures in persons with PTSD to exploring the therapeutic potential of SCFAs, prebiotics, and novel psychobiotics, the research presented here underscores the dynamic interplay between the gut and the brain. By integrating diverse methodologies, including systematic reviews, clinical trials, preclinical studies, and Mendelian randomization, we gain insights into the complex mechanisms underlying PTSD. Ultimately, this Research Topic of studies advocates for a paradigm shift in PTSD management, emphasizing a holistic approach that considers the gut microbiome as a key player in mental health and paves the way for innovative, personalized interventions that may transform the lives of those suffering from the enduring effects of trauma.

